# Transcriptome profiling of aerial and subterranean peanut pod development

**DOI:** 10.1038/s41597-024-03205-3

**Published:** 2024-04-11

**Authors:** Zhenying Peng, Kai-Hua Jia, Jingjing Meng, Jianguo Wang, Jialei Zhang, Xinguo Li, Shubo Wan

**Affiliations:** Key Laboratory of Crop Genetic Improvement & Ecology and Physiology, Institute of Crop Germplasm Resources, Shandong Academy of Agricultural Sciences, Jinan, 250100 China

**Keywords:** Plant evolution, Plant development

## Abstract

Peanut (*Arachis hypogaea*) showcases geocarpic behavior, transitioning from aerial flowering to subterranean seed development. We recently obtained an atavistic variant of this species, capable of producing aerial and subterranean pods on a single plant. Notably, although these pod types share similar vigor levels, they exhibit distinct differences in their physical aspects, such as pod size, color, and shell thickness. We constructed 63 RNA-sequencing datasets, comprising three biological replicates for each of 21 distinct tissues spanning six developmental stages for both pod types, providing a rich tapestry of the pod development process. This comprehensive analysis yielded an impressive 409.36 Gb of clean bases, facilitating the detection of 42,401 expressed genes. By comparing the transcriptomic data of the aerial and subterranean pods, we identified many differentially expressed genes (DEGs), highlighting their distinct developmental pathways. By providing a detailed workflow from the initial sampling to the final DEGs, this study serves as an important resource, paving the way for future research into peanut pod development and aiding transcriptome-based expression profiling and candidate gene identification.

## Background & Summary

Angiosperm fruiting is divided into four modes according to the spatial location of the fruit: aerocarpy, basicarpy, geocarpy, and amphicarpy^1,2^. Aerocarpy exists in most plants, with the fruit developing on aboveground reproductive branches. Basicarpy refers to plants whose flowers (including ovaries) and fruit are produced close to ground level, which is mostly observed in trailing or creeping plants. Geocarpy refers to the development of fruit below ground, and amphicarpy describes plants that develop fruits both above and below ground^3–5^.

Geocarpy and amphicarpy are rare fruiting modes that mainly occur in herbaceous plants growing in habitats lacking water or light, or those subject to frequent soil disturbance or severe environmental fluctuations^1,3^. These two fruiting modes are important ecological adaptations^5,6^, with geocarpy allowing plants to preserve offspring in a suitable microenvironment near the mother plant, maintain seed vitality under extreme environments, avoid herbivores and fire damage^1,3^. Geocarpy is often thought to be an ‘*in situ* adaptation’ that occurs in response to dramatic climate change^1,3^. The mechanisms by which geocarpy occurs and its evolution are yet to be elucidated.

Geocarpy is generally considered to have evolved from aerocarpy through amphicarpy, the likely intermediate evolutionary stage between the two^1,3^. Amphicarpic characteristics may be an adaptive bet-hedging strategy in response to dramatic environmental changes^1^. Soil protects subterranean seeds from heat, cold, drought, and predators, whereas aerial seeds are better able to disperse from the mother plant and potentially establish new habitats^7^. In amphicarpic plants, the early production of subterranean seeds almost guarantees reproduction, and the later production of aerial seeds increases the reproductive ability at the end of plant growth^8^. Considering the different characteristics of the two seed types, amphicarpy offers plants a greater fitness advantage than geocarpy for coping with environmental changes. Amphicarpic plants adjust the ratio of aerial and subterranean seeds in response to their environment, thus increasing the viability of their progeny.

Peanut (*Arachis hypogaea*) is a classic geocarpic plant with aerial flowers and subterranean seeds. After blooming, the fertilized ovary of a sessile chasmogamous flower penetrates the soil via an elongated ‘peg’, and its tip quickly develops into a subterranean pod^9,10^. If the pegs cannot penetrate the soil, the embryo cannot develop into a pod (early embryo abortion)^9,10^. Whether pegs develop into pods depends on a variety of factors, with mechanical stimulus and/or darkness being essential conditions^11^. In our preliminary study, we developed a peanut variety named ‘Shunhua 25’ that produces aerial pods (50 or more) and subterranean pods (20–30). Notably, this peanut variety does not require mechanical stimuli or darkness to produce pods. The aerial pods are small, with a green shell and a shorter development period than the subterranean pods; however, seedlings grown from aerial seeds show the same reproductive ability as those derived from subterranean seeds. This atavistic peanut variety is an excellent material for studying the mechanisms of pod development in geocarpic and amphicarpic plants.

In this study, we conducted transcriptome analyses of the aerial and subterranean pods across six developmental stages (S1–S6; defined below), encompassing components such as pegs, underground and aboveground shells, kernels, and seed coats. We describe in detail the construction of 63 RNA-sequencing (RNA-seq) libraries, which resulted in 409.36 Gb of clean bases obtained using transcriptome analysis pipelines consisting of quality control, quantification, and differential gene expression analyses. A principal components analysis and a hierarchical clustering of gene expression data were used to infer the quality of the RNA-seq data and the characteristics of each sample. The extensive transcriptome data will provide valuable information for future studies of the peanut pod development mechanism.

## Methods

### Plant materials and categorization of development

**Fig. 1:**
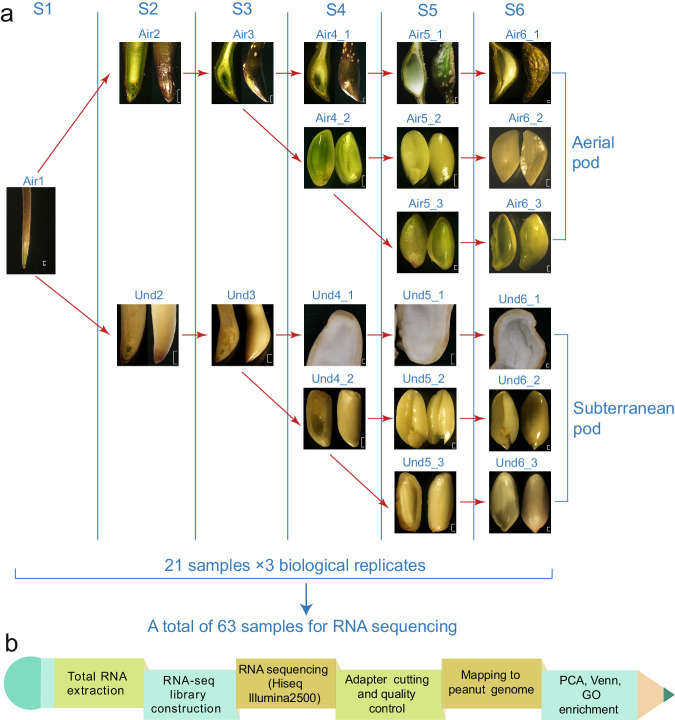
Sampling and sequencing for the peanut aerial and subterranean pods. (**a**) The six developmental stages **(**S1–S6) of peanut aerial (Air) and subterranean (Und) pods. Air1 (S1) refers to the peg rather than a pod, and is a shared stage in both aerial and subterranean development. The S2 and S3 stages include a blend of pod shell, kernel, and seed coat samples, Air2 and Air3 for aerial pod, while Und2 and Und3 for subterranean pod. From the S4 stage onward, these tissues were sampled separately. Air4_1 and Air4_2 correspond to the aerial pod shell, and a combination of the kernel and seed coat, respectively; while Und4_1 and Und4_2 to the subterranean pod shell, and a combination of the kernel and seed coat, respectively. S5 stage referred to the immature pod, Air5_1, Air5_2 and Air5_3 correspond to the aerial pod shell, seed kernel and seed coat, respectively; while Und5_1, Und5_2 and Und5_3 correspond to the aerial pod shell, seed kernel and seed coat, respectively. S6 stage referred to the mature pod, Air6_1, Air6_2 and Air6_3 correspond to the aerial pod shell, seed kernel and seed coat, respectively; while Und6_1, Und6_2 and Und6_3 correspond to the aerial pod shell, seed kernel and seed coat, respectively. (**b**) Simplified pipeline of the RNA-seq process.

The peanut (*A. hypogaea*) variety ‘Shunhua 25’ was cultivated in the Yinmaquan experimental base in Jinan, China (N36°39′2.81″, E117°06′49.95″). Pod development was categorized into six stages based on the characteristics of the shells and seeds of both aerial and subterranean pods (Fig. 1a). These stages were labeled Air1 to Air6 for the aerial pods, and Air1 to Und6 for the subterranean pods.

All newly formed pegs were considered to be the initial common stage (Air1) for both pod types. At this stage (S1), the pegs were slender, with color variations along their length. The next stage (S2) saw the pegs develop into pods, with aerial pods (Air2) showing green coloration and swelling at the tips, whereas the subterranean pods (Und2) turned white after penetrating the soil. As development continued (S3), the pods became more swollen and smooth, displaying color changes and forming a spongy tissue inside (Air3 and Und3). Stage 4 (S4) was characterized by the development of a reticulated (net-like pattern) shell and a thickened spongy tissue in the pods (Air4_1 and Und4_1), with small embryos present inside the seeds (Air4_2 and Und4_2). The fifth stage (S5) saw further shell development and the growth of the seeds, with immature embryonic lobules (Air5_1, Air5_2 and Air5_3, Und5_1, Und5_2 and Und5_3). By the final stage (S6), the pod reached maturity, with a dark-green (aerial) or light-yellow (subterranean) shell, fully developed seeds, and mature embryonic lobules (Air6_1, Air6_2 and Air6_3, Und6_1, Und6_2 and Und6_3).

### RNA extraction, library construction, and sequencing

RNA was extracted from the 21 samples, each with three biological replicates. The entire process, from RNA extraction to data analysis, is depicted in Fig. 1b.

RNA was isolated using the TRIzol reagent (Thermo Fisher Scientific, Waltham, MA, USA) and treated with RNase-free DNase I (New England Biolabs, Ipswich, MA, USA) at 37 °C for 30 min to eliminate any contaminating DNA. The concentration and purity of the resulting RNA samples were evaluated using a NanoDrop 2000 spectrophotometer (Thermo Fisher Scientific), and the integrity was assessed using an RNA Nano 6000 Assay Kit (Agilent Technologies, Santa Clara, CA, USA).

A 1.5-μg RNA aliquot was subjected to rRNA removal using the Epicentre Ribo-Zero rRNA Removal Kit (Illumina, San Diego, CA, USA), and the remaining RNA was used to prepare sequencing libraries employing the NEBNext Ultra Directional RNA Library Prep Kit for Illumina (New England Biolabs). Index codes were incorporated to assign each sequence to its respective organ of provenance. Paired-end sequences were generated using the Illumina Hiseq 2500 platform.

The raw RNA-seq data were processed for quality control using fastp v0.12.4^12^, which removed the low-quality bases and adapter sequences. After filtering, the trimmed reads were evaluated and the high-quality results were merged using multiQC v1.13.dev0^13^ with default parameters. The sequences were mapped to the peanut reference genome^14^ using hisat2 v2.2.1^15^. The featureCounts v2.0.1^16^ program was used to obtain the raw read counts, which were normalized to quantify the expression abundances of the transcripts using the transcripts per million (TPM) value as the measure. A principal component analysis (PCA) of the TPM across all samples was performed using the prcomp function from the stats package in R v4.2.0^17^. The differential expression analysis was conducted using DESeq 2 v1.34.0^18^. Genes with an adjusted *p*-value < 0.05 and a |fold change| (|FC|) > 2 between different samples were considered to be differentially expressed genes (DEGs). Utilizing the pheatmap package in R, the expression patterns of the top 500 DEGs for each paired combination of samples were identified and visually represented in a heatmap. Venn diagrams were constructed using the VennDiagram package^19^ in R. A gene ontology (GO) enrichment analysis was performed using the clusterProfiler package^20^ in R, with the significance criteria set at *p < *0.05 and an adjusted *p*-value (*p*adj) < 0.05.

### Identification of DEGs between aerial and subterranean pods

**Fig. 2:**
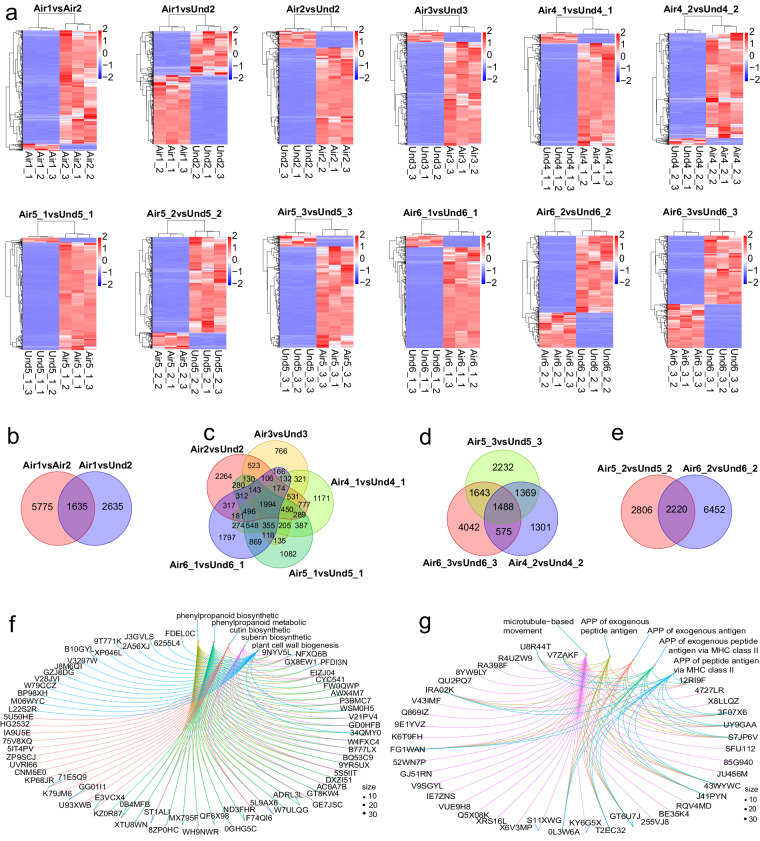
Expression profiles and DEGs in response to each paired combination. (**a**) Expression patterns of the top 500 DEGs for each paired combination. Upregulated DEGs are highlighted in red; downregulated DEGs are shown in blue. DEGs were defined using an adjusted *p*-value < 0.05 and |FC| > 2. (**b–e**) Venn diagrams of the number of unique and shared DEGs between (**b**) Air1vsAir2 and Air1vsUnd2; (**c**) Air2vsUnd2, Air3vsUnd3, Air4_1vsUnd4_1, Air5_1vsUnd5_1, and Air6_1vsUnd6_1; (**d**) Air4_2vsUnd4_2, Air5_3vsUnd5_3, and Air6_3vsUnd6_3; and (**e**) Air5_2vsUnd5_2 and Air6_2vsUnd6_2. (**f**) DEGs involved in the top five GO terms represented in the shared DEGs between the paired combinations Air1vsAir2 and Air1vsUnd2 (Fig. 2b). (**g**) DEGs involved in the top five GO terms represented in the shared DEGs between the paired combinations Air5_2vsUnd5_2 and Air6_2vsUnd6_2 (Fig. 2e). APP is short for ‘antigen processing and presentation’.

To investigate the developmental differences between the aerial and subterranean pods, we performed 12 paired comparisons: Air1 vs. Air2 to identify DEGs between the pegs and aerial pods; Air1 vs. Und2 to identify DEGs between the pegs and underground pods; Air2 vs. Und2 and Air3 vs. Und3 to identify DEGs between the developed aerial and underground pods; Air4_1 vs. Und4_1 to identify DEGs between the immature pod shells of aerial and subterranean pods; Air4_2 vs. Und4_2 to identify DEGs between the immature seed coats of aerial and subterranean pods; Air5_1 vs. Und5_1 to identify DEGs between the moderately mature pod shells of aerial and subterranean pods; Air5_2 vs. Und5_2 to identify DEGs between the moderately mature kernels of aerial and subterranean pods; Air5_3 vs. Und5_3 to identify DEGs between the moderately mature seed coats of aerial and subterranean pods; Air6_1 vs. Und6_1 to identify DEGs between the mature pod shells of aerial and subterranean pods; Air6_2 vs. Und6_2 to identify DEGs between the mature kernels of aerial and subterranean pods; and Air6_3 vs. Und6_3 to identify DEGs between the mature seed coats of aerial and subterranean pods. The top 500 DEGs for each paired comparison were visualized in heatmaps (Fig. 2a). All of the DEGs can be accessed on figshare (10.6084/m9.figshare.23633835)^21^.

**Fig. 3:**
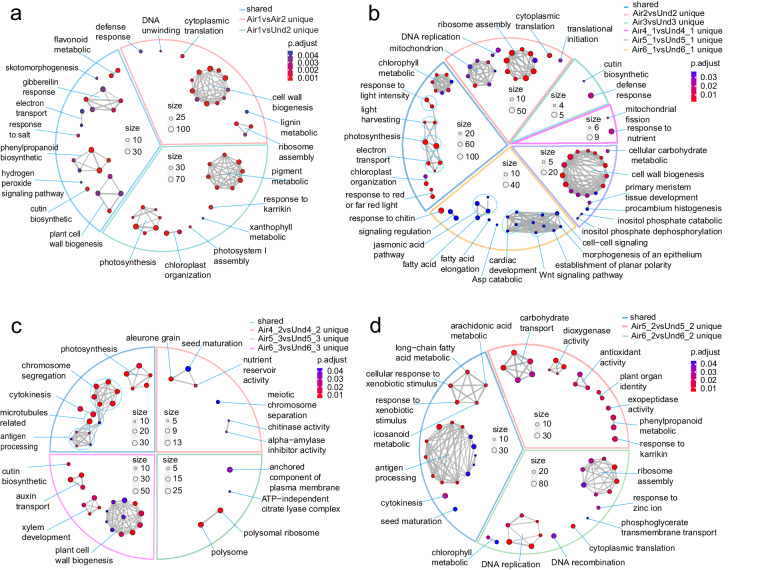
Top 20 GO terms of the shared and unique DEGs for each paired combination. Top 20 biological process GO terms of the shared and unique DEGs in Fig. 2b (**a**), Fig. 2c (**b**), Fig. 2d (**c**), and Fig. 2e (**d**).

We constructed four Venn diagrams to elucidate the unique and shared DEGs from the combination pairs (Fig. 2b–e). The first Venn diagram aimed to discern DEGs pertinent to the developmental initiation of pegs in aerial and subterranean pods, specifically in Air1 vs. Air2 and Air1 vs. Und2. This analysis resulted in 5,775 and 2,635 unique DEGs in Air1 vs. Air2 and Air1 vs. Und2, respectively, of which 1,635 were shared between the two comparisons (Fig. 2b). A cnetplot was used to explore the top five GO terms of the 1,635 shared DEGs, revealing that 35 of these DEGs were involved in phenylpropanoid biosynthesis, 36 in phenylpropanoid metabolism, 18 in cutin biosynthesis, 16 in suberin biosynthesis, and 17 in plant cell wall biogenesis (Fig. 2f). The shared DEGs were enriched in GO pathways, such as phenylpropanoid biosynthesis, the gibberellin response, and plant cell wall biosynthesis (Fig. 3a). The DEGs unique to the Air1 vs. Air2 comparison were enriched in functions associated with cell wall biogenesis, ribosome assembly, and cytoplasmic translation, whereas the Air1 vs. Und2 unique DEGs were enriched in pigment metabolism, photosynthesis, and chloroplast organization (Fig. 3a). These findings indicate that, in the initial stage of peg development, the main differences between aerial and subterranean pegs are concentrated in cell wall formation and photosynthetic pigment biosynthesis.

The second Venn diagram analyzed gene expression in peanut pod shells across five combinations: Air2 vs. Und2, Air3 vs. Und3, Air4_1 vs. Und4_1, Air5_1 vs. Und5_1, and Air6_1 vs. Und6_1. We identified 2,264, 766, 1,171, 1,082, and 1,791 unique DEGs in each paired comparison, respectively, along with 1,994 shared DEGs across multiple combinations (Fig. 2c). The shared DEGs were enriched in GO pathways such as photosynthesis, chlorophyll metabolism, and the response to light intensity (Fig. 3b). The Air2 vs. Und2 unique DEGs were enriched in functions associated with ribosome assembly, mitochondrion biology, and DNA replication, whereas the Air3 vs. Und3 unique DEGs were enriched in the defense response and cutin biosynthesis. The Air4_1 vs. Und4_1 unique DEGs were enriched in functions associated with the response to nutrients and mitochondrial fission, whereas the Air5_1 vs. Und5_1 unique DEGs were enriched in plant cell wall biogenesis and cellular carbohydrate metabolism. The Air6_1 vs. Und6_1 unique DEGs were enriched in Wnt signaling pathway and fatty acid functions. The unique DEGs of each paired combination are therefore clearly distinct from each other.

The third Venn diagram, related to the peanut seed coat, encompassed three paired combinations (Air4_2 vs. Und4_2, Air5_3 vs. Und5_3, and Air6_3 vs. Und6_3). We identified 2,232, 1,301, and 4,042 unique DEGs, including 1488 shared DEGs (Fig. 2d). The 1,488 shared DEGs were enriched in GO pathways such as microtubule-related processes, photosynthesis, and chromosome segregation (Fig. 3c). The Air4_2 vs. Und4_2 unique DEGs were enriched in functions associated with seed maturation, aleurone grains, and nutrient reservoir activity, whereas the Air5_3 vs. Und5_3 unique DEGs were enriched in polysomal ribosome functions, anchored components of the plasma membrane, and the ATP-independent citrate lyase complex. The Air6_3 vs. Und6_3 unique DEGs were enriched in plant cell wall biogenesis, auxin transport, and xylem development functions. The significant differences between these paired combinations centered on the cell wall biogenesis and photosynthesis activities.

The fourth Venn diagram, focusing on the peanut seed kernels, involved two paired combinations (Air5_2 vs. Und5_2 and Air6_2 vs. Und6_2), from which 2,806 and 6,452 unique DEGs were identified, respectively, of which 2,220 were shared (Fig. 2e). The 2,220 shared DEGs were enriched in GO pathways such as microtubule-based movement, several antigen processes, and cytokinesis (Fig. 3d). The DEGs of the top five GO annotations were parsed using a cnetplot diagram (Fig. 2g), revealing 38, 13, 13, 13, and 13 shared DEGs associated with microtubule-based movement, antigen processing and presentation of exogenous peptide antigens, antigen processing and presentation of exogenous antigens, antigen processing and presentation of exogenous peptide antigens via MHC class II, and antigen processing and presentation of peptide antigens via MHC class II, respectively. The Air5_2 vs. Und5_2 unique DEGs were enriched in functions associated with carbohydrate transport, dioxygenase activity, and antioxidant activity, whereas the Air6_2 vs. Und6_2 unique DEGs were enriched in ribosome assembly, DNA replication, and chlorophyll metabolism.

## Data Records

The RNA-seq reads, derived from 63 samples encompassing both aerial and subterranean pods, have been consigned to the National Center for Biotechnology Information (NCBI) Sequence Read Archive (SRA) database under the accession number SRP448232^22^. In addition, the TPM data, count data, DEGs, heatmap visualizing the DEGs, GO enrichment are available from the figshare repository (10.6084/m9.figshare.23633835)^21^.

## Technical Validation

### Quality control

**Fig. 4:**
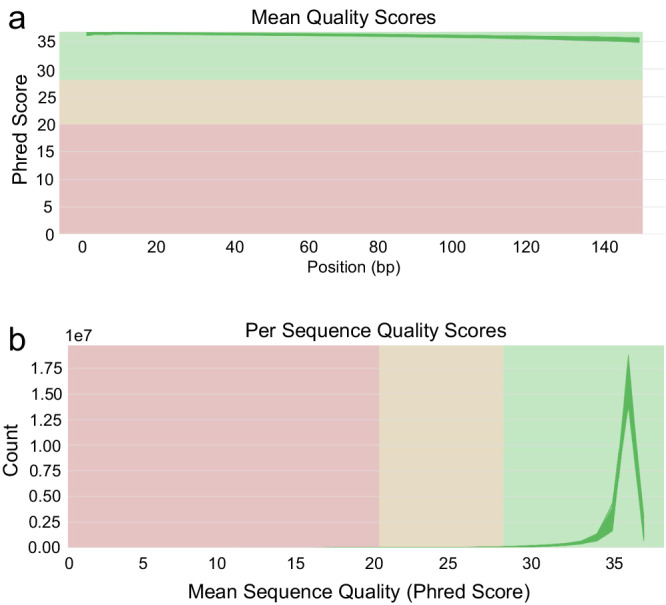
Quality assessment of the RNA-sequencing data. (**a**) Mean quality scores per sample. (**b**) Per-sequence quality scores of the individual samples.

We assessed the quality of the RNA-seq data by examining the average quality score per position and per sequence using multiQC^13^ as shown in Fig. 4. The quality score for all sequences exceeded 30 (Fig. 4a), and the distribution of per-sequence quality scores was predominantly within the 30–40 range (Fig. 4b), confirming the high quality of the reads.

### Analysis of transcriptome data

**Fig. 5:**
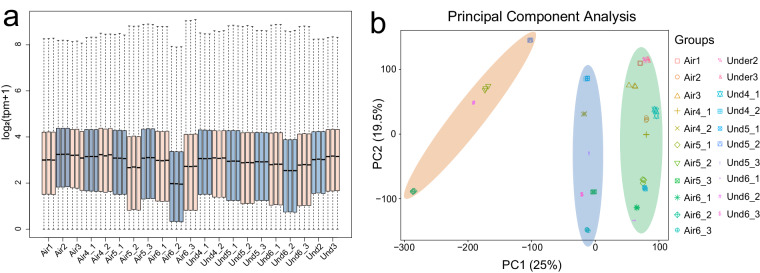
Global assessment of the transcriptome data. (**a**) Expression levels across different samples. (**b**) PCA based on TPM. The manually drawn ellipses in various colors represent the clustering of samples.

The transcriptome analysis of the 63 samples yielded 409.36 Gb of clean bases. These preprocessed reads were aligned to the *A. hypogaea* reference genome using hisat2 v2.2.1^15^, achieving an average mapping rate of 92.07%. We used boxplot graphs to display the distribution of gene expression levels of the samples (Fig. 5a). The similarity in the distribution between sample repeats underscores the high consistency of our data.

We performed PCA on the RNA-seq data derived from distinct tissues of the aerial and subterranean pods. The results classified the 21 sample types into three distinct clusters. The first cluster (depicted by the green ellipse in Fig. 5b) encompasses the 11 samples (Air1, Air2, Air3, Air4_1, Air5_1, Air6_1, Und2, Und3, Und4_1, Und5_1, and Und6_1) representing the shell parts of the peanut pod. The second cluster (illustrated by the blue ellipse in Fig. 5b) comprises the six samples (Air4_2, Air5_3, Air6_3, Und4_2, Und5_3, and Und6_3) that correspond to the seed coat of the peanut pod. The third cluster (marked by the orange ellipse in Fig. 5b) includes the four samples (Air5_2, Air6_2, Und5_2, and Und6_2) that represent the seed kernel of the peanut pod. Samples from the same and similar tissues were clustered together, showing similar patterns, which further indicates the reliability of our data.

## Data Availability

Software and their versions used for RNA-seq analysis were described in Methods. No custom code was used to generate or process the data described in the manuscript.
